# Recognition and Accommodation at the Androgen Receptor Coactivator Binding Interface

**DOI:** 10.1371/journal.pbio.0020274

**Published:** 2004-08-24

**Authors:** Eugene Hur, Samuel J Pfaff, E. Sturgis Payne, Hanne Grøn, Benjamin M Buehrer, Robert J Fletterick

**Affiliations:** **1**Graduate Group in Biophysics, University of CaliforniaSan Francisco, California, United States of America; **2**Karo Bio, DurhamNorth Carolina, United States of America; **3**Department of Biochemistry and Biophysics, University of CaliforniaSan Francisco, CaliforniaUnited States of America

## Abstract

Prostate cancer is a leading killer of men in the industrialized world. Underlying this disease is the aberrant action of the androgen receptor (AR). AR is distinguished from other nuclear receptors in that after hormone binding, it preferentially responds to a specialized set of coactivators bearing aromatic-rich motifs, while responding poorly to coactivators bearing the leucine-rich “NR box” motifs favored by other nuclear receptors. Under normal conditions, interactions with these AR-specific coactivators through aromatic-rich motifs underlie targeted gene transcription. However, during prostate cancer, abnormal association with such coactivators, as well as with coactivators containing canonical leucine-rich motifs, promotes disease progression. To understand the paradox of this unusual selectivity, we have derived a complete set of peptide motifs that interact with AR using phage display. Binding affinities were measured for a selected set of these peptides and their interactions with AR determined by X-ray crystallography. Structures of AR in complex with FxxLF, LxxLL, FxxLW, WxxLF, WxxVW, FxxFF, and FxxYF motifs reveal a changing surface of the AR coactivator binding interface that permits accommodation of both AR-specific aromatic-rich motifs and canonical leucine-rich motifs. Induced fit provides perfect mating of the motifs representing the known family of AR coactivators and suggests a framework for the design of AR coactivator antagonists.

## Abstract 2

A structural study of the androgen receptor shows how it interacts with coactivator proteins. The results also provide a possible framework for the development of new receptor antagonists

## Introduction

The androgen receptor (AR) is the cellular mediator of the actions of the hormone 5-α dihydrotestosterone (DHT). Androgen binding to AR leads to activation of genes involved in the development and maintenance of the male reproductive system and other tissues such as bone and muscle. However, it is the pivotal role of AR in the development and progression of prostate cancer that has led to increasing interest in this nuclear receptor. Presently, hormone-dependent prostate cancer is treated with a combination of strategies that reduce circulating levels of androgens, such as the administration of antiandrogens that compete for the androgen-binding pocket in the core of the C-terminal ligand-binding domain (LBD). The benefits of these treatments are typically transient, with later tumor growth associated with increases in expression levels of AR or its cofactors, or mutations that render AR resistant to antiandrogens (Gregory et al. 2001; Culig et al. 2002; Lee and Chang 2003). Alternative approaches to inhibiting AR transcriptional activity may therefore lie in disrupting critical protein associations the receptor needs for full function.

The precise details of how AR binds the dozens of coregulator proteins reported to associate with different regions of AR in vivo remain poorly understood (Lee and Chang 2003). Many nuclear receptors activate transcription by binding short leucine-rich sequences conforming to the sequence LxxLL (where “x” is any amino acid), termed nuclear receptor (NR) boxes, which are found within a variety of NR coactivators including the p160 family. Hormone binding to the LBD stabilizes the C-terminal helix of the receptor, helix 12, in a conformation that completes a binding surface for these LxxLL motifs (Darimont et al. 1998; Nolte et al. 1998; Shiau et al. 1998; Bledsoe et al. 2002). The structural elements composing this binding interface, consisting of helices 3, 4, 5, and 12 of the receptor, are synonymous with a previously defined hormone-dependent activation function that lies within the LBD termed activation function (AF)–2. Association of p160 coactivators allows the recruitment and assembly of a number of other cofactors that together modulate the state of chromatin and interactions with components of the basal transcription machinery to initiate transcription (Glass and Rosenfeld 2000).

AR, however, utilizes multiple mechanisms to activate gene transcription. Generally, AR activity is dependent on contributions from multiple transactivation functions that lie within the N-terminal domain (NTD) collectively called AF-1. Although the AR AF-2 can bind to a restricted set of LxxLL motifs (Ding et al. 1998; He et al. 1999; Needham et al. 2000) and is relatively potent (Wang et al. 2001), it usually displays weak independent activity at typical androgen-regulated genes, with significant activity observed only in the presence of high levels of p160 coactivators, as detected in some prostate cancers (He et al. 1999; Gregory et al. 2001). Instead, the AR AF-2 exhibits a distinct preference among NRs for phenylalanine-rich motifs conforming to the sequence FxxLF (He et al. 2000; He and Wilson 2003). Such motifs have been identified in the AR NTD and in an AR cognate family of coactivators that includes AR-associated protein (ARA) 54, ARA55, and ARA70 (He et al. 2000, 2002b; Lee and Chang 2003). The NTD FxxLF motif (residues 23–27) mediates a direct, interdomain, ligand-dependent interaction between the NTD and LBD (N/C interaction) that is thought to facilitate dimerization, stabilize androgen binding, and possibly regulate AF-1 and AF-2 activity (Langley et al. 1998; He et al. 2000). In addition, the NTD also contains a related hydrophobic motif, WxxLF (residues 433–437), that nucleates formation of an alternative N/C interaction that may serve to inhibit AR activity (He et al. 2000, 2002a; Hsu et al. 2003).

Presently, how the AR AF-2 surface can accommodate residues with bulky aromatic side chains and distinguish FxxLF motifs from LxxLL motifs is not known. To understand the structural basis of this unusual coactivator recognition preference, we characterized the full repertoire of interacting sequences using phage display to define amino acids preferred at the AR coactivator binding interface. Crystal structures of the AR LBD in complex with several phage display–derived peptides reveal the structural basis of FxxLF motif specificity and an induced fit of the receptor that allows accommodation of other related hydrophobic motifs. Comparisons of the structures suggest strategies for the design of AR coactivator antagonists.

## Results

### AR Preference for Aromatic Groups in Coregulator Recognition

**Figure 1:**
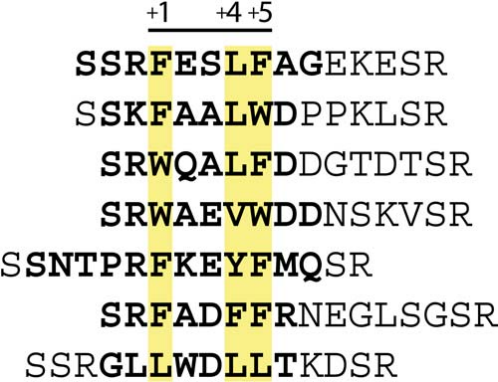
AR LBD–Interacting Peptides Selected by Phage Display Hydrophobic residues of the core motif are highlighted in yellow. Residues in bold were ordered in electron density maps.

Phage display has been used to study coactivator recognition specificity and to identify coactivator motif sequence variants preferred by the estrogen receptor (ER), thyroid hormone receptor (TR) β, and most recently AR (Chang et al. 1999; Norris et al. 1999; Paige et al. 1999; Northrop et al. 2000; Hsu et al. 2003). Using phage display, we screened more than 2 × 10^10^ randomized peptides against DHT-bound AR LBD. Selections identified sequences containing hydrophobic motifs that were primarily aromatic in character, consistent with another recent study (Hsu et al. 2003) (Figure 1). Of these aromatic motifs, FxxLF and related motifs with substitutions of phenylalanine or tryptophan for leucine at positions +1, +5, or both, dominated the selections. (Peptide residues are numbered in reference to the first hydrophobic residue of the core motif, which is numbered +1. Residues preceding the first hydrophobic residue are numbered negatively in descending order starting with −1.) Substitutions of tyrosine at the +5 position were also observed, but to a much lesser extent (unpublished data). At the +4 position, valines, methionines, and even the aromatic residues phenylalanine and tyrosine were observed (Figure 1; unpublished data). In general, LxxLL motifs were not selected. The LxxLL motif shown in Figure 1 was derived from prior phage selections with ER and subsequently demonstrated to bind AR in FRET-based screens in vitro (unpublished data).

Preliminary characterization of the subset of AR-interacting peptides shown in Figure 1 confirmed that each competed for binding of in vitro translated AR cofactors to bacterially expressed AR LBD in pulldown assays, and generally did so with modestly improved efficiency relative to the native FxxLF motif from the AR NTD and significantly greater efficiency than a native LxxLL motif from glucocorticoid receptor-interacting protein 1 (GRIP1) NR box 3 (P. Webb, personal communication). The equilibrium dissociation constants *(K_d_)* were directly determined for the interaction between the AR LBD and FxxLF and LxxLL peptides and one variant tryptophan-containing peptide, FxxLW, using surface plasmon resonance (Table 1). The *K_d_* for FxxLF was 1.1 μM, similar to the affinities of physiologically derived FxxLF motifs determined previously by isothermal titration calorimetry (He and Wilson 2003). The affinity of LxxLL was less than 2-fold weaker, with a *K_d_* of 1.8 μM, but more than three times stronger than the tightest binding p160-derived LxxLL motif, NR box 3 of transcriptional intermediary factor 2 (TIF2) (He and Wilson 2003). Surprisingly, the affinity of FxxLW, with a *K_d_* of 920 nM, was slightly better than FxxLF, in spite of the presence of the tryptophan residue at the +5 position. Together, our results are consistent with the notion that the phage display peptides interact with the same AR surface that binds FxxLF and LxxLL motifs in native cofactors, and that they do so with similar or improved affinities relative to their natural counterparts.

**Table 1:**
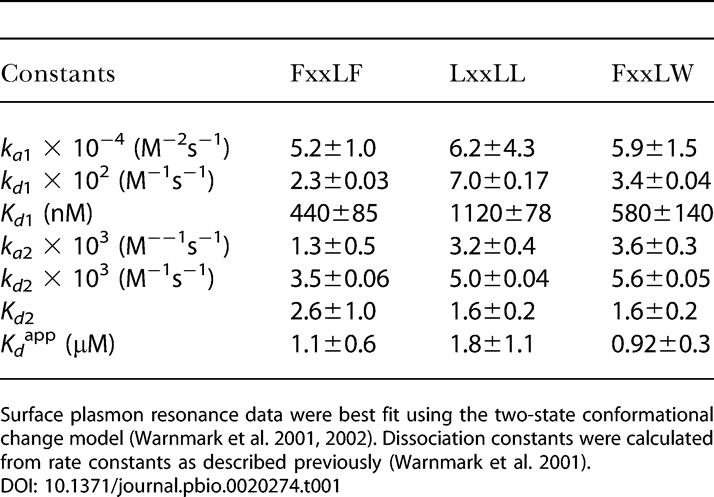
Rate and Dissociation Constants for the Interaction between the AR LBD and Selected Peptides

Surface plasmon resonance data were best fit using the two-state conformational change model (Warnmark et al. 2001, 2002). Dissociation constants were calculated from rate constants as described previously (Warnmark et al. 2001)

### One Site Fits All

**Figure 2:**
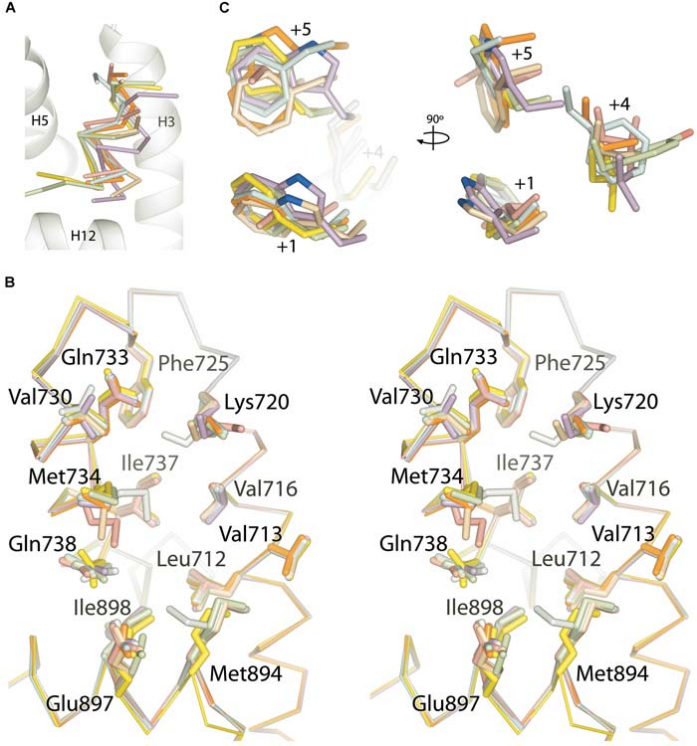
A Structural Profile of the AR Coactivator Binding Interface AR–peptide complexes are colored as follows: FxxLF, yellow; FxxLW, orange; WxxLF, wheat; WxxVW, purple; FxxYF, green; FxxFF, blue; LxxLL, pink; unbound, grey. (A) Cα trace of the peptides superimposed onto the AF-2. For clarity only the LBD of AR–FxxLF is shown. (B) Superposition of the LBD of the AR–peptide complexes in the region of the coactivator interface. Backbone atoms are shown as a Cα trace. Side chains of residues composing the interface are shown as sticks. (C) Hydrophobic side chains of the core motif superimposed as in (B).

To understand the binding mode of different AR coactivators, we determined the crystal structures of DHT-bound AR LBD without peptide and in complex with each of the seven peptides listed in Figure 1. All complexes crystallized in the space group *P*2_1_2_1_2_1_ with one molecule per asymmetric unit and unit cell dimensions similar to those observed in previous AR LBD crystal structures (Matias et al. 2000; Sack et al. 2001). Overall structural features of the complexes are shown in Figure 2. Peptides assumed short α helical conformations centered on the core hydrophobic motif and bound in a solvent channel relatively free of crystal contacts on a groove formed by helices 3, 4, 5, and 12 of the receptor (Figure 2A). Detailed data collection and refinement statistics, as well as buried surface areas for each complex, are listed in Table 2. The structures confirm previous suggestions that AR utilizes a single binding interface for LxxLL and noncanonical aromatic-rich motifs (He et al. 2000, 2002a). Only side chains move to accommodate the array of peptides, sometimes considerably, with the unbranched side chains of Lys720, Met734, and Met894 making the largest conformational changes upon binding of peptide (Figure 2B).

**Table 2:**
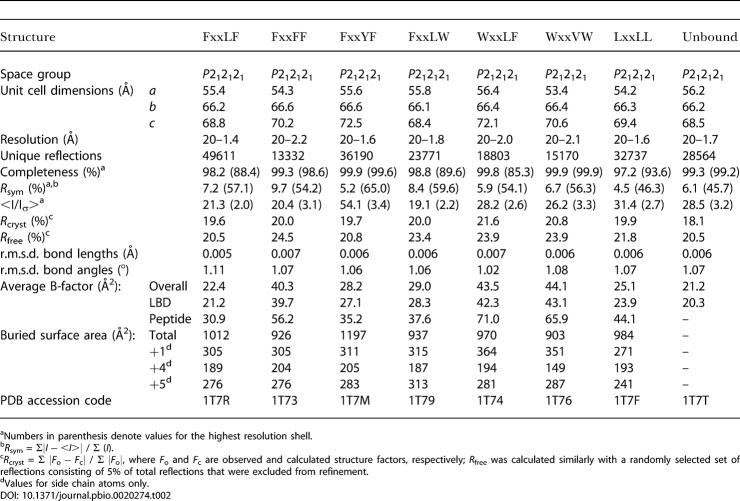
Summary of Structures and Crystallographic Statistics

^a^Numbers in parenthesis denote values for the highest resolution shell

^b^ *R*_sym_ = Σ|*I* − <*I*>| / Σ (*I*)

^c^ *R*_cryst_ = Σ |*F*_o_ − *F*_c_| / Σ |*F*_o_|, where *F*_o_ and *F*_c_ are observed and calculated structure factors, respectively; *R*_free_ was calculated similarly with a randomly selected set of reflections consisting of 5% of total reflections that were excluded from refinement

^d^Values for side chain atoms only

### FxxLF

**Figure 3:**
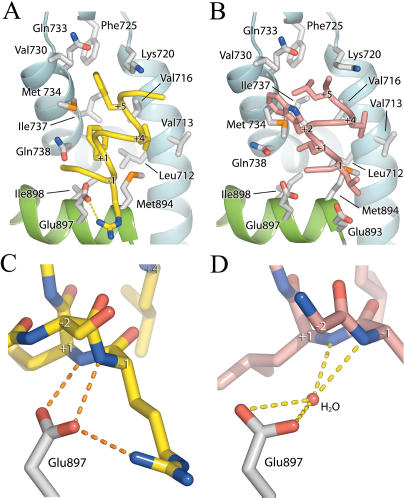
Interactions of FxxLF and LxxLL with the AR LBD (A and B) FxxLF (A) and LxxLL (B) bound to the AR AF-2 interface. FxxLF and LxxLL are shown as yellow and pink Cα coils, respectively. Helices 3, 4, and 5 of the LBD are shown as blue ribbons; Helix 12 is shown in green. LBD residues interacting with peptides are depicted as white sticks. For clarity only peptide side chains making significant interactions with the LBD are shown. (C and D) Hydrogen-bonding interactions between backbone atoms of FxxLF (C) and LxxLL (D) with Glu897 of the LBD. Peptide alpha carbons are labeled.

The mechanisms that permit AR to accommodate motifs with bulky phenylalanine residues were assessed in a crystal structure of the AR LBD in complex with the FxxLF peptide. The FxxLF peptide recapitulates the binding mode of p160-derived LxxLL motifs to other nuclear receptors (Darimont et al. 1998; Nolte et al. 1998; Shiau et al. 1998; Bledsoe et al. 2002). The peptide forms a short α helix whose hydrophobic face, composed of Phe+1, Leu+4, and Phe+5, binds an L-shaped groove formed by helices 3, 4, 5, and 12 of the LBD that is composed of three subsites that accommodate each hydrophobic residue (Figures 2A and 3A). The conserved charged residues at either end of the cleft, Lys720 and Glu897, the so-called charge clamp residues, make electrostatic interactions with the main chain atoms at the ends of the peptide helix: Lys720 with the carbonyl group of Phe+5, and Glu897 with the amide nitrogens of Phe+1 and Arg−1 (Figure 3C). Glu897 also interacts with the side chain of Arg−1. The two interior residues of the motif, Glu+2 and Ser+3, are solvent exposed and do not interact with the receptor.

**Figure 4:**
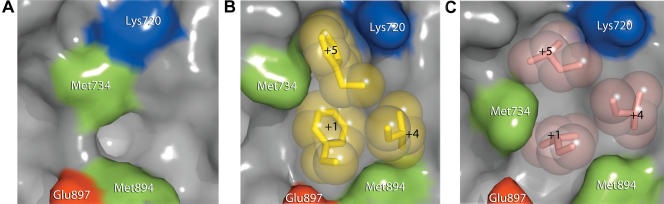
Induced Fit of the AR AF-2 Interface Surface representations of the AR AF-2 interface. The unbound structure is shown in (A), the FxxLF bound in (B), and the LxxLL bound in (C). Side chains of the hydrophobic residues of the core motifs of FxxLF and LxxLL are shown as spheres.

Comparison of AR alone and AR in complex with FxxLF (and other aromatic-rich peptides described below) reveals that the AF-2 cleft reorganizes to accommodate the bulky peptide side chains (see Figures 2B and 4). The unbranched side chains of Lys720 and Met734 move from an extended conformation over the +5 pocket to one almost perpendicular to the surface of the protein. The pockets for Phe+1 and Phe+5 are arranged in a line, forming a deep, extended cleft on the LBD spanning the length of the two side chains on the face of the peptide helix (see Figures 3A and 4B). Phe+1, almost entirely solvent inaccessible, binds face down at the base of this groove, making hydrophobic contacts with Leu712, Val716, Met734, Gln738, Met894, and Ile898, which define the +1 pocket. The top of the groove, composed of Val716, Lys720, Phe725, Ile737, Val730, Gln733, and Met734, narrows to form the +5 pocket. Met734 and the aliphatic portion of Lys720 constrict this subsite, forming van der Waals interactions with opposite faces of the Phe+5 benzyl ring. Together, the +1 and +5 residues are almost entirely solvent inaccessible. In contrast, Leu+4 binds in a shallow hydrophobic patch consisting of Leu712 and Val716 lined at the ridges by Val713 and Met894 and is largely solvent exposed.

### LxxLL

The preference of AR for motifs with aromatic groups over leucine-rich motifs was assessed with a crystal structure of the AR LBD in complex with the LxxLL peptide. The structure reveals similarities between the binding modes of the LxxLL and FxxLF motifs to AR, and other LxxLL motifs to other nuclear receptors. The LxxLL motif adopts a helical conformation, and interactions of the motif with the AF-2 cleft are predominantly hydrophobic, with the three leucine residues of the motif contributing most of the interactions. However, significant differences can be seen between the binding mode of the LxxLL motif to AR and that of p160-derived LxxLL motifs to other nuclear receptors. First, flanking residues were largely disordered, with only two N-terminal flanking residues and one C-terminal residue visible in electron density maps (see Figures 1 and 3B). This contrasts with extended structures seen in the p160-derived LxxLL motifs in complex with their cognate receptors (Darimont et al. 1998; Nolte et al. 1998; Shiau et al. 1998; Bledsoe et al. 2002). Second, the LxxLL peptide backbone forms hydrogen bonds with only one of the two conserved charge clamp residues, Lys720. A shift in the position of the LxxLL peptide helix precludes direct interactions with Glu897 (see Figures 2A and 3D). This shift results from changes in the geometry of the +1 and +5 subsites mediated by Met734, which moves 2.5Å toward the +1 pocket (see Figures 2B and 4C) and enables binding of a leucine at the +5 subsite by a simultaneous widening and shallowing of the pocket. This movement of Met734 causes displacement of the +1 residue, resulting in a rotation of the peptide helix away from helix 12, toward helix 3. A slight translation of the peptide helix also occurs away from helix 12 because of the shorter side chain length of leucine (see Figure 2A).

Side chains of residues flanking the first leucine of the motif make additional hydrophobic interactions with the AR surface (see Figure 3B). Trp+2 reaches over Met734, clamping the methionine in between itself and Leu+1. Leu−1 extends over Met894, abutted against Glu893. These interactions likely explain the moderate affinity of AR for this particular LxxLL motif despite suboptimal complimentarity with the residues of the core motif (as discussed below) and the loss of main chain interactions with Glu897.

### WxxLF, FxxLW, and WxxVW

**Figure 5:**
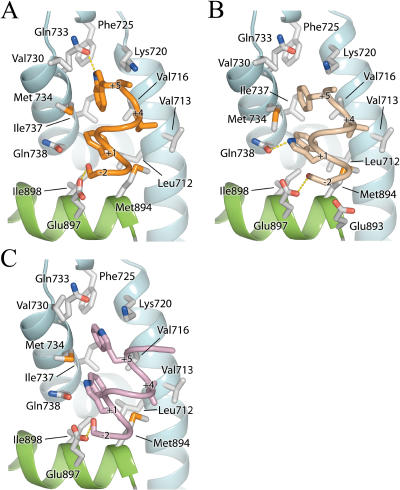
Interactions of the Tryptophan Motifs with the AR LBD FxxLW (A), WxxLF (B), and WxxVW (C) bound to the AR AF-2 interface. FxxLW, WxxLF, and WxxVW are shown as orange, beige, and purple Cα coils, respectively. The LBD is depicted as in Figure 3.

**Figure 6:**
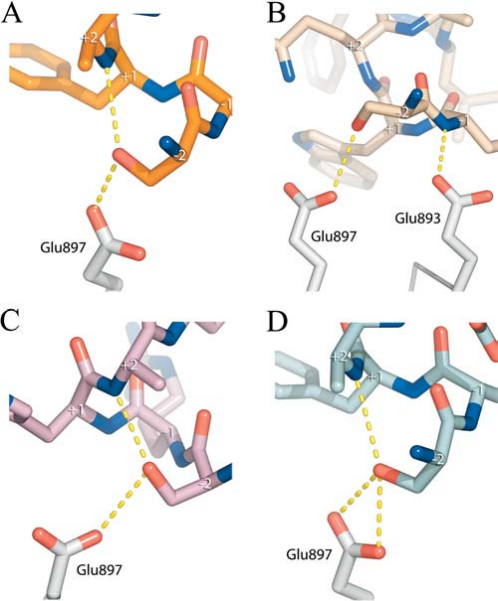
Interactions of Ser−2 with Glu897 Interactions between Ser−2 of the peptides (A) FxxLW, (B) WxxLF, (C) WxxVW, and (D) FxxFF and Glu897 of the LBD. Peptide alpha carbons are labeled.

To understand how the AR AF-2 accommodates tryptophan residues, structures of AR in complex with peptides containing tryptophan substitutions at the +1 or +5 position, or both, were determined (Figure 5). Surprisingly, WxxLF, analogous to the only tryptophan-containing motif known in vivo, WHTLF in the AR NTD, was relatively disordered, with the peptide displaying the highest B-factor and least well defined density, suggesting that it binds with the lowest affinity (Table 2). Nonetheless, each of the tryptophan peptides adopted similar helical conformations. As described above for the LxxLL motif, substitutions at the +1 and +5 positions for non-phenylalanine residues result in shifts of the peptide helix (see Figure 2A). Consequently, backbone interactions with Lys720 are maintained, but interactions with the other charge clamp residue, Glu897, are lost. Once again, however, flanking residues within the peptide make additional contacts with the AR surface, and, unlike the LxxLL peptide, these contacts include Glu897. In FxxLW and WxxVW, the −2 serine (Figure 6) forms a bidentate hydrogen-bonding interaction, making hydrogen bonds to both Glu897 and the backbone amide group of the +2 residue. Ser−2 of WxxLF similarly interacts with Glu897, but is too distant for helical-capping interactions with the +2 amide group. Instead, Glu893, in a more typical interaction with the +1 amide nitrogen, caps the WxxLF helix (Figure 6B). Thus, tryptophan substitutions are tolerated, but they induce a shift in the peptide backbone that precludes interactions with one of the charge clamp residues. This suboptimal interaction is compensated partially by interactions of flanking residues with the AR surface.

### FxxFF and FxxYF

**Figure 7:**
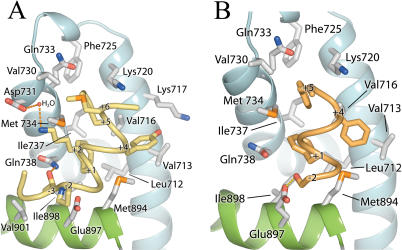
Interactions of FxxYF and FxxFF with the AR LBD FxxYF (A) and FxxFF (B) bound to the AR AF-2 interface. FxxYF and FxxFF are shown as yellow and orange Cα coils, respectively. The LBD is depicted as in Figure 3.

Finally, effects of substitutions at the +4 position were assessed in structures of AR in complex with peptides containing FxxFF and FxxYF motifs (Figure 7). Surprisingly, the binding mode of FxxFF to AR resembled that of the tryptophan peptides more closely than the binding mode of FxxLF (see Figures 2A and 7B). Like the tryptophan peptides, interactions with Glu897 are mediated by Ser−2 instead of the peptide backbone (see Figure 6D). Deviations from ideal helical geometry allow Phe+4 to bind facedown in the +4 pocket with the benzyl ring stacked against Val713.

**Figure 8:**
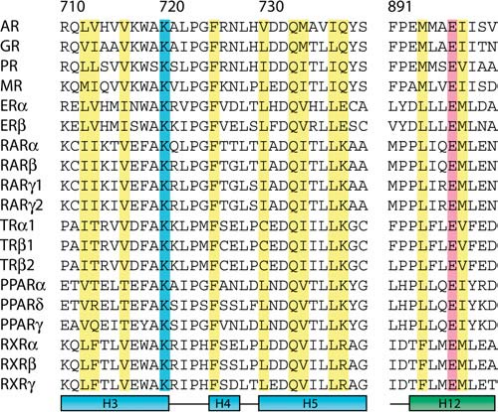
Sequence Alignment of the AF-2 Region of NRs Residues composing the coactivator interface of AR are highlighted in yellow. The absolutely conserved glutamate and lysine composing the charge clamp are highlighted in pink and blue, respectively. Residue numbering is that of AR.

By contrast, the conformation of FxxYF was the closest to FxxLF (see Figure 2A). Other than FxxLF, only FxxYF makes direct backbone interactions with Glu897. Unlike the facedown orientation of Phe+4 observed in the FxxFF peptide, Tyr+4 is bound edgewise into the shallow +4 pocket, making interactions with Val713, Val716, and the aliphatic portion of Lys717. FxxYF was the most ordered of all the peptides, with 12 out of 15 residues observed in the electron density (see Figures 1 and 7A). Significant interactions were observed involving residues other than hydrophobic residues of the motif. Lys+2 and Met+6 are predominantly solvent exposed, extending out over the protein surface. Met+6 is bound on top of Phe+5, while Lys+2 makes a water-mediated hydrogen bond with Asp731. Thr−3 of the peptide defines a new subsite, with the hydroxyl group forming a hydrogen bond to Gln738 and the methyl group making hydrophobic contacts in a pocket formed by Glu897, Ile898, and Val901. Similar interactions were observed in the glucocorticoid receptor (GR)–TIF2 complex involving the −3 glutamine of the TIF2 NR box 3 motif (Bledsoe et al. 2002). However a valine to asparagine substitution at the residue corresponding to 901 in AR creates a pocket with a more polar character in GR (Figure 8).

### Restrictions of the Three Subsites

Together, the structures described above permit an assessment of the way that individual subsites of the AR AF-2 cleft accommodate hydrophobic groups. The indole rings of tryptophan and the phenyl rings of phenylalanine fit into their pockets analogously with the +1 and +5 residues bound facedown and edgewise, respectively, into the AF-2 cleft. On the other hand, the position of the +4 residue is variable, with binding in this shallow pocket largely dictated by the position of the peptide backbone caused by the bound conformations of the +1 and +5 residues (see Figure 2C). Small shifts in the position of the N-terminal of helix 12 can be seen, which reposition Met894 for more optimal contacts with +4 residues bound at that subsite (see Figure 2B).

The binding mode detected in the +1 pocket is the most conserved of the three hydrophobic subsites (see Figure 2C). The benzyl moiety of the indole side chains superimpose with the corresponding benzyl side chains of the phenylalanine-rich motifs, effectively mimicking interactions of a phenylalanine residue. However, the presence of a hydrogen-bonding partner on the indole side chain enables an additional polar interaction not seen in the phenylalanine-rich motifs between the indole nitrogen and Gln738 (see Figure 5B). Unexpectedly, this additional interaction in the +1 pocket does not occur with Trp+1 of WxxVW (see Figure 5C). While similarly distanced to make the same interaction, the plane of the indole ring is rotated about 20° relative to that of WxxLF, causing it to be at a poor angle for strong hydrogen bonding to Gln738.

Binding of tryptophans in the +5 pocket is slightly more variable (see Figure 2C). Trp+5 of WxxVW is bound similarly to phenylalanine residues at the same position. Only the six-membered ring of the indole group is fully buried in the pocket. The five-membered ring of the indole side chain sticks out, solvent exposed. In contrast, the +5 indole group of FxxLW is rotated almost 90°, resulting in burial of both rings of the indole group, as well as the formation of a strong hydrogen bond between the indole nitrogen and Gln730 (see Figure 5A). Binding in this orientation appears to be highly favorable, as the FxxLW peptide deviates from helical geometry at the +5 position to do so.

## Discussion

**Figure 9:**
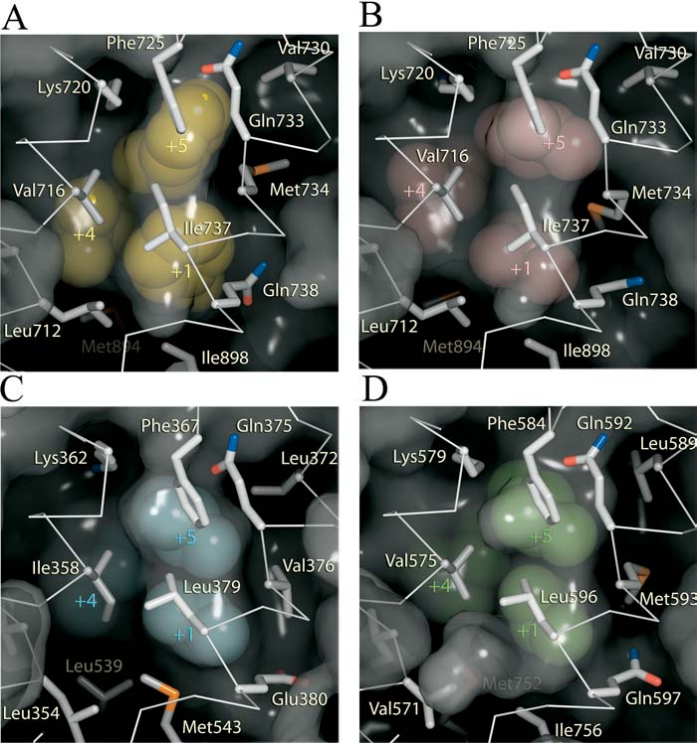
Surface Complimentarity of Hydrophobic Motifs in the AR, ERα, and GR AF-2 Clefts (A) AR–FxxLF, (B)AR–LxxLL, (C) ERα–GRIP1 (LxxLL) (Shiau et al. 1998), and (D) GR-TIF2 (LxxLL) (Bledsoe et al. 2002). The inside surfaces of the AF-2 cleft in AR, ERα, and GR are depicted. The LBD is additionally shown as a Cα trace with key side chains shown as white sticks. Phenylalanines and leucines of the FxxLF and LxxLL motifs are shown as spheres.

The crystal structures reported here reveal how AR binds coactivator motifs with bulky aromatic hydrophobic groups and permit construction of a profile of the AR coregulator interface (see Figure 2). In some ways, this interface resembles those of other nuclear receptors: it is an L-shaped hydrophobic cleft comprised of three distinct subsites that bind hydrophobic groups at the +1, +4, and +5 positions in cognate peptides. Moreover, the so-called charge clamp residues (Lys720 and Glu897) bracket the cleft. Nonetheless, the AR coregulator recognition site is unique in that it rearranges upon motif binding to form a long, deep, and narrow groove that accommodates aromatic residues at the +1 and +5 positions (Figure 9). Sequence alignments of AR with other NRs suggest that a unique combination of substitutions at Val730, Met734, and Ile737 combine to permit the formation of a smoother, flatter interaction surface that displays a higher complimentarily to aromatic substituents than to branched aliphatic (see Figure 8). Of these, methionine, the only unbranched hydrophobic amino acid and the most accommodating, at a key position between the +1 and +5 sites, allows the AR AF-2 interface to vary the size and shape of its pockets to associate with a more diverse set of coregulators. GR also contains a methionine residue at this position, raising the possibility that it may also employ induced fit to broaden motif recognition. While naturally occurring mutations in AR have yet to be observed at Met734, it is interesting to note that mutations at Val730 and Ile737 have been reported in patients with prostate cancer and androgen insensitivity, respectively (Newmark et al. 1992; Quigley et al. 1995; Gottlieb et al. 1998).

The same characteristics that make the AR AF-2 ideal for binding of longer, aromatic side chains also make it less well suited for binding of shorter, branched side chains. Although changes in the position of Met734 widen the groove towards the +5 subsite to permit binding of leucine residues, the gross features of the groove remain largely the same (see Figure 9B). As a result, the +1 and +5 leucines bind in a smooth, elongated groove and interactions between the +1 and +5 residues on the face of the peptide helix, or with a hydrophobic “bump” present in other receptors caused by a isoleucine to leucine substitution between the +1 and +5 subsites, are absent. Thus, a smaller proportion of the available surface area is available for van der Waals interactions.

Unlike the conserved interaction modes of aromatic residues with the +1 and +5 sites, binding interactions at the +4 site are variable and characterized by nonspecific interactions. This finding agrees with the relatively high conservation of residues at the +1 and +5 positions of AR-interacting motifs and suggests that these residues drive peptide interaction with the LBD, whereas the +4 site is less critical. Indeed, the +4 pocket is shallow, surface exposed, and relatively featureless, explaining the assortment of residues selected at the +4 position. It is likely that any hydrophobic residue that does not clash with surrounding residues would be suitable at this subsite.

While peptide motif recognition is governed by hydrophobic interactions, polar interactions from backbone atoms and residues outside the core motif also contribute. With the exception of FxxFF, motifs containing phenylalanines at the +1 and +5 positions present canonical main chain interactions with both charge clamp residues, Lys720 and Glu897. This finding stands in contrast to predictions of previous studies (Alen et al. 1999; He et al. 1999; Slagsvold et al. 2000; He and Wilson 2003), which concluded that Lys720 was dispensable for FxxLF binding and that Glu897 was required for binding to FxxLF and LxxLL motifs. Lys720 comprises a significant portion of the +5 subsite, making important van der Waals interactions with the Phe+5 benzyl group in addition to hydrogen bonds to the motif backbone. These results suggest that Lys720 is required for binding of FxxLF motifs. However, it may be that enough binding energy is provided by the other residues of the +5 subsite (i.e., Met734), as well as by the other subsites themselves, such that removal of Lys720 would have little effect on binding. Observations that Lys720 plays a greater role in LxxLL motif binding are likely due to the fact that there is less surface area contributing to van der Waals contacts in LxxLL motifs. Disrupting binding contributions from Lys720 would thus have a more detrimental effect on binding.

On the other hand, Glu897 interacts with the FxxLF peptide backbone, but is disengaged from the LxxLL peptide backbone. One possible explanation for the apparent requirement for Glu897 in LxxLL binding is that it might interact with residues outside of the core motif. The corresponding glutamate of GR, Glu 755, forms hydrogen bonds with the −3 asparagine of TIF2 NR box 3 (Bledsoe et al. 2002), and Glu897 of AR participates in noncanonical interactions with the hydroxyl group of a Ser−2 residue that was selected in all of our tryptophan-containing peptides. This is especially intriguing given that the only WxxLF motif known in vivo, located in the AR NTD, also possesses a Ser−2 residue. WxxLF also makes backbone interactions with an alternate charge clamp residue, Glu893, pointing towards adaptability in AR AF-2 charge clamp formation.

Sequence alignment of NR coactivator sequences shows that positively charged residues are favored N-terminal to the core hydrophobic motif while negatively charged residues are favored C-terminal to the motif (He and Wilson 2003). Our phage-selected peptides are consistent with this trend. Arginines and lysines were observed at the N-terminal −1 position in all peptides, except for LxxLL, in which Arg was present at the −3 position. Moreover, four out of seven peptides contained negatively charged aspartate or glutamate residues C-terminal to the core motif. While previous studies have shown that complementary interactions between charged residues flanking coactivator signature motifs of coactivators and charged residues surrounding the AF-2 cleft modulated binding to the receptor (He and Wilson 2003), we find that the flanking charged residues are typically disordered in the electron density, with only Arg−1 of FxxLF interacting with Glu897, and Lys+2 of FxxYF forming a water-mediated hydrogen bond to Asp731. Thus, if charge–charge interactions between flanking peptide residues and the AR surface occur, they are too weak to be detected crystallographically.

Finally, the AR AF-2 surface is an attractive target for pharmaceutical design. Selective peptide inhibitors that bind the AF-2 surface of liganded ERα, ERβ, and TRβ have been developed (Geistlinger and Guy 2003), and similar α-helix–mediated protein–protein interfaces have successfully been targeted with tight binding small molecule inhibitors (Asada et al. 2003; Vassilev et al. 2004). Drugs that directly interfere with coactivator binding or formation of the AR N/C interaction would likely inhibit AR activity, perhaps even in androgen-resistant prostate cancers in which conventional therapies have failed. Strategies for designing AR coactivator antagonists are revealed in spite of the changes to the structure at the interface. Together the +1, +4, and +5 subsites contribute the majority of buried surface area of the peptide–LBD interaction (Table 2). Inhibitors may be designed by varying hydrophobic constituents at these hotspots. The +1 and +5 subsites of AR have a unique preference for aromatic side chains and provide the most viable starting points for designing AR-specific inhibitors. Aromatic groups, possibly with polar constituents to exploit hydrogen bonding interactions with Gln733 and Gln738 in the +1 and +5 subsites, respectively, may provide promising leads. Indeed, initial screens have yielded compounds that bind to the +1 subsite in such a manner (E. Estébanez-Perpiñá, personal communication). Poorly conserved binding and a lack of strong structural features at the +4 subsite suggest that this site may be incorporated for achieving other characteristics important for inhibitors besides fit. Synthetic strategies that link together groups that bind with moderate affinity to the +1, +5, and possibly +4 subsites may yield tight binding inhibitors of AR coactivator association.

## Materials and Methods

### 

#### Protein purification

Expression and purification of the AR LBD for crystallization were performed essentially as described (Matias et al. 2000). The cDNA encoding the chimp AR LBD (residues 663–919—human numbering), which displays 100% identity to the human form in protein sequence, was cloned into a modified pGEX-2T vector (Amersham Biosciences, Piscataway, New Jersey, United States) and expressed as glutathione S-transferase (GST) fusion protein in the E. coli strain BL21 (DE3) STAR in the presence of 10 μM DHT. Induction was carried out with 30 μM IPTG at 17 °C for 16–18 h. E. coli cells were lysed in buffer (10 mM Tris, [pH 8.0], 150 mM NaCl, 10% glycerol, 1 mM TCEP, 0.2 mM PMSF) supplemented with 0.5 μg/ml lysozyme, 5 U/ml benzonase, 0.5% CHAPS, and 10 μM DHT. All buffers for further purification steps contained 1 μM DHT. Soluble cell lysate was adsorbed to Glutathione Sepharose 4 Fast Flow resin (Amersham Biosciences), washed with buffer containing 0.1% n-octyl β-glucoside, and eluted with 15 mM glutathione. After cleavage of the GST moiety with thrombin, final purification of the AR LBD was carried out using a HiTrap SP cation exchange column (Amersham Biosciences). Eluted AR LBD was dialyzed overnight at 4 °C against buffer containing 50 mM HEPES (pH 7.2), 10% glycerol, 0.2 mM TCEP, 20 μM DHT, 150 mM Li_2_SO_4_, and 0.1% n-octyl β-glucoside, then concentrated to greater than 4 mg/ml for crystallization.

Purification of AR LBD for use in phage affinity selection was carried out as above without the final dialysis and concentration steps. The expression construct contained the AR LBD as an inframe fusion with GST in a modified pGEX-2T vector containing both a flexible region and an AviTag sequence (Avidity, Denver, Colorado, United States) allowing in vivo biotinylation. The GST–AR LBD fusion expression plasmid was cotransformed with a plasmid-encoding E. coli biotin ligase (Avidity) into BL21 (DE3) STAR cells. Protein expression was carried out as above but with induction supplemented with 50 μM biotin to ensure quantitative biotinylation of AR LBD.

#### Phage affinity selections and peptide identification

Phage affinity selections were performed essentially as described (Paige et al. 1999). Biotinylated AR LBD (10 pmol/well) was incubated in streptavidin-coated Immulon 4 96-well plates (Dynatech International, Edgewood, New Jersey, United States) in TBST (10 mM Tris-HCl [pH 8.0], 150 mM NaCl, 0.05% Tween 20) with 1 μM DHT for 1 h at 4 °C. Affinity selections were performed in TBST containing 1 μM DHT. M13 phage distributed among 24 libraries displaying a total of greater than 2 × 10^10^ different random or biased amino acid sequences were added to the wells containing immobilized AR LBD and incubated for 3 h at 4 °C. After washing, bound phage were eluted using pH 2 glycine. Enrichment of phage displaying target-specific peptides was monitored after each round of affinity selection using an anti-M13 antibody conjugated to horseradish peroxidase in an ELISA–type assay.

Synthetic peptides corresponding to the deduced amino acid sequences from receptor-specific phage were tested for their ability to interact with purified AR LBD using a FRET–based assay format. Peptides were synthesized according to the deduced amino acid sequence displayed on phage with an additional C-terminal amino acid sequence consisting of SGSGK to allow the attachment of a biotin tag (Anaspec, San Jose, California, United States). Flourophor conjugates were prepared by incubating either biotinylated peptides with streptavidin-cryptate (Cis Bio International, Bagnols Sur Ceze Cedex, France), or biotinylated AR LBD with streptavidin-XL665 (Cis Bio). Interaction between peptide and AR LBD was monitored by the ratio of energy transfer by excitation at 320 nm and emission at 625 nm and 665 nm.

#### Surface plasmon resonance

Affinities of peptides to the AR LBD were determined with a Biacore (Piscataway, New Jersey, United States) 2000 instrument. A peptide derived from silencing mediator for RXR and TR 2 (SMRT2) served as a negative control. 1 mM peptide stock solutions in DMSO were diluted into HBS-P buffer (10 mM HEPES [pH 7.4], 150 mM NaCl, 0.005% Surfactant P20) to generate 10 μM working solutions. HBS-P buffer was flowed through the cells to achieve a stable baseline prior to immobilization of the biotinylated peptides. To achieve the binding of approximately 250 RU of peptides to individual cells, working solutions of peptides were diluted to 100 nM in HBS-P buffer. Unbound streptavidin sites were blocked by injection of a 1 mM biotin solution at a rate of 10 μl/min.

Purified AR LBD was diluted into HBS-P buffer to a concentration of 10 μM and injected into all four Flowcells using the Kinject protocol at a flow rate of 10 μl/min (contact time 360 s, dissociation time 360 s). Following the dissociation phase, the surface of the chip was regenerated to remove residual AR LBD by QuickInject of buffer containing 10 mM HEPES and 50% ethylene glycol (pH 11). Following the establishment of a stable baseline, the same procedure was repeated using a series of AR LBD dilutions (5 μM, 1 μM, and 300 nM) in an iterative manner. Analysis of the data was performed using BIAevaluation 3.0 software (Biacore). The SMRT2 signals were subtracted as background from the three remaining peptide signals. Data were best fit using the two-state conformational change model (Warnmark et al. 2001, 2002).

#### Crystallization, data collection, and refinement

Purified, concentrated AR LBD was combined with 3x to 6x molar excess of peptide and incubated 1 h at room temperature before crystallization trials. Complexes were crystallized using the hanging drop vapor diffusion method. Protein–peptide solution was combined in a 1:1 ratio with a well solution consisting of 0.6–0.8 M sodium citrate and 100 mM Tris or HEPES buffer (pH 7–8). Crystals typically appeared after 1–2 d, with maximal size attained within 2 wk. For data collection, crystals were swiped into a cryo-protectant solution consisting of well solution plus 10% glycerol before flash freezing in liquid nitrogen. The addition of ethylene glycol to a well concentration of 10%–20% was later found to both improve crystal quality and enable the freezing of crystals directly out of the drop.

Datasets were collected at 100K at the Advanced Light Source (Lawrence Berkeley Laboratory, Berkeley, California, United States), beamline 8.3.1, with either a ADSC Quantum 315 or Quantum 210 CCD detector. Data were processed using Denzo and Scalepack (Otwinowski and Minor 1997). Molecular replacement searches were performed with rotation and translation functions from CNS (Brunger et al. 1998). Initial searches for AR–FxxLF were performed using the structure of AR–R1881 (PDB: 1E3G) with R1881 omitted from the search model. Subsequent searches for all other complexes were performed using the refined LBD structure from the AR–FxxLF complex. To minimize the possibility of model bias, FxxLF peptide and DHT were omitted from all molecular replacement searches. Protein models were built by iterative rounds of simulated annealing, conjugate gradient minimization, and individual B-factor refinement in CNS followed by manual rebuilding in Quanta 2000 (Accelrys, San Diego, California, United States) using σ_A_-weighted 2*F*_o_ − *F*_c_, *F*_o_ − *F*_c_, and simulated annealing composite omit maps. Superposition of structures was performed with LSQMAN (Kleywegt 1996). Buried surface area calculations were performed with CNS. All figures were generated with PyMOL (DeLano 2002). Coordinates and structure factors for all complexes have been deposited in the Protein Data Bank. Accession numbers are listed in Table 2.

## Supporting Information

### Accession Numbers

The Swiss-Prot (http://www.ebi.ac.uk/swissprot) accession numbers for the gene products discussed in this paper are AR (P10275), ARA54 (Q9UBS8), ARA55 (Q9Y2V5), ARA70 (Q13772), ER (P03372, Q92731), glucocorticoid receptor-interacting protein 1 NR box 3 (Q61026 ), GR (P04150), NR box 3 of TIF2 (Q15596), and TR β (P10828).

The Protein Data Bank (http://www.rcsb.org/pdb) accession numbers for the structures used in this paper are FxxFF (1T73), FxxLF (1T7R), FxxLW (1T79), FxxYF (1T7M), LxxLL (1T7F), unbound (1T7T), WxxLF (1T74), and WxxVW (1T76).

## Abbreviations

AF: activation function
AR: androgen receptor
ARA: androgen receptor-associated protein
DHT: 5-α dihydrotestosterone
ER: estrogen receptor
FxxFF: phenylalanine-x-x-phenylalanine-phenylalanine
FxxLF: phenylalanine-x-x-leucine-phenylalanine
FxxLW: phenylalanine-x-x-leucine-tryptophan
FxxYF: phenylalanine-x-x-tyrosine-phenylalanine
GR: glucocorticoid receptor
GRIP1: glucocorticoid receptor-interacting protein 1
GST: glutathione S-transferase
*K_d_*: equilibrium dissociation constant
LBD: ligand-binding domain
LxxLL: leucine-x-x-leucine-leucine
N/C interaction: interaction between the N-terminal domain and the ligand-binding domain
NR: nuclear receptor
NTD: N-terminal domain
SMRT2: silencing mediator for RXR and TR 2
TIF2: transcriptional intermediary factor 2
TR: thyroid hormone receptor
WxxLF: tryptophan-x-x-leucine-phenylalanine
WxxVW: tryptophan-x-x-valine-tryptophan
